# Biochemical and Molecular Phylogenetic Study of Agriculturally Useful Association of a Nitrogen-Fixing Cyanobacterium and Nodule *Sinorhizobium* with *Medicago sativa* L.

**DOI:** 10.1155/2015/202597

**Published:** 2015-05-31

**Authors:** E. V. Karaushu, I. V. Lazebnaya, T. R. Kravzova, N. A. Vorobey, O. E. Lazebny, D. A. Kiriziy, O. P. Olkhovich, N. Yu. Taran, S. Ya. Kots, A. A. Popova, E. Omarova, O. A. Koksharova

**Affiliations:** ^1^Educational and Scientific “Institute of Biology”, Taras Shevchenko National University of Kyiv, 64/13 Volodymyrska Street, Kyiv 01601, Ukraine; ^2^N. I. Vavilov Institute of General Genetics, Russian Academy of Sciences, Gubkin Street 3, Moscow 119333, Russia; ^3^Lomonosov Moscow State University, Biocenter, Leninskie Gory 1-12, Moscow 119991, Russia; ^4^Institute of Plant Physiology and Genetics, National Academy of Sciences of Ukraine, 31/17 Vasylkivska Street, Kyiv 03022, Ukraine; ^5^N. K. Kol'tsov Institute of Developmental Biology, Russian Academy of Sciences, Vavilova Street 26, Moscow 119334, Russia; ^6^Institute of Molecular Genetics, Russian Academy of Sciences, Moscow 123182, Russia; ^7^Lomonosov Moscow State University, Belozersky Institute of Physical-Chemical Biology, Leninskie Gory 1-40, Moscow 119992, Russia

## Abstract

Seed inoculation with bacterial consortium was found to increase legume yield, providing a higher growth than the standard nitrogen treatment methods. Alfalfa plants were inoculated by mono- and binary compositions of nitrogen-fixing microorganisms. Their physiological and biochemical properties were estimated. Inoculation by microbial consortium of *Sinorhizobium meliloti* T17 together with a new cyanobacterial isolate *Nostoc* PTV was more efficient than the single-rhizobium strain inoculation. This treatment provides an intensification of the processes of biological nitrogen fixation by rhizobia bacteria in the root nodules and an intensification of plant photosynthesis. Inoculation by bacterial consortium stimulates growth of plant mass and rhizogenesis and leads to increased productivity of alfalfa and to improving the amino acid composition of plant leaves. The full nucleotide sequence of the rRNA gene cluster and partial sequence of the dinitrogenase reductase (*nifH*) gene of *Nostoc* PTV were deposited to GenBank (JQ259185.1, JQ259186.1). Comparison of these gene sequences of *Nostoc* PTV with all sequences present at the GenBank shows that this cyanobacterial strain does not have 100% identity with any organisms investigated previously. Phylogenetic analysis showed that this cyanobacterium clustered with high credibility values with *Nostoc muscorum*.

## 1. Introduction

Continuous anthropogenic impact on the environment of different chemicals, fertilizers, herbicides, plant protection from pests and diseases, plant growth regulators, and so forth that are used in agriculture, makes it necessary to develop an alternative to agricultural production, which would be based on the use of cost-effective and environmentally friendly systems for land application of fertilizers and plant protection. An important role in this respect is given to the maximum use of the soil microflora.

In many countries around the world, studies and implementation of the compositions consisting of symbiotic and free-living nitrogen-fixing microorganisms have started to increase productivity of crops. Among the wide range of diazotrophic microorganisms cyanobacteria are the most versatile for biochemical potential, since they do not need to be provided with soil organic substances for nitrogen fixation unlike heterotrophic nitrogen-fixing microorganisms.

Positive ecological role of cyanobacteria in the soil as nitrogen-fixing bacteria which participate in deposition of organic matter is well known now, and besides they are the centers of microcosms as autotrophic organisms with amazing abilities for symbiotrophic relations [1, 2]. The last property of cyanobacteria is particularly interesting in case of using the consortia of microorganisms in biotechnology instead of monocultures [3]. In nature, cyanobacteria are never found in the form of cell populations of one species. They are in a close relationship with the microbial community, located in the mucus of the surrounding cells. Research in this field has shown that the composition of satellite cyanobacteria is very labile and it depends on changes in habitat conditions. Axenic cultures of cyanobacteria exist only in the laboratories. In nature, they form a community and, being the edificators of microbial communities, cyanobacteria can change the microbial composition [4]. It allows the constructing of artificial microbial consortium. Nitrogen-fixing activity (NFA) of soil compositions of diazotrophic microorganisms can be an effective way to supply the crop by environmentally friendly biological nitrogen. Use of this approach requires in-depth study of the relationship between bacteria, cyanobacteria, and plants, as well as compatibility of microorganisms-partners in created artificial associations. It is important to perform the screening of the most suitable strains of microorganisms, to create conditions for the effective functioning of these symbiotic consortia. It is necessary to select an optimal quantitative ratio of microorganisms and methods of their implantation into the rhizosphere.

The goals of this research were the study of the effect of artificial stable microbial consortium based on nitrogen-fixing cyanobacterium* Nostoc* PTV and Tn5-mutant of nodule bacteria* Sinorhizobium meliloti* T17, on the physiological and biochemical characteristics of growth and development of alfalfa, and, finally, on its yield and product quality and the molecular typing and phylogenetic analysis of this new cyanobacterial isolate* Nostoc* PTV.

## 2. Material and Methods

### 2.1. Organisms and Growth Conditions

Plant alfalfa* Medicago sativa* (L.) sort of Jaroslavna obtained from the NSC Institute of Agriculture of National Academy of Agrarian Sciences of Ukraine has been used in the experiments. For the inoculation of alfalfa seeds we used the strain of nodule bacteria* Sinorhizobium* (*Rhizobium*)* meliloti* T17 (patent of Ukraine number 55432) from the collection of nitrogen-fixing microorganisms of the Institute of Plant Physiology and Genetics, National Academy of Sciences of Ukraine (Kyiv) [6]. The strain of* S. meliloti* T17 was obtained as a result of intergeneric conjugation of* Escherichia coli* S17-1 (pSUP2021::Tn5) and* S. meliloti* 425a on agar medium TY (tryptone/yeast extract) as described in [7] and it was selected for improved symbiotic properties. To create the binary composition of nitrogen-fixing microorganisms the culture of cyanobacterium* Nostoc* PTV (from the collection of the Institute of Hydrobiology, National Academy of Sciences of Ukraine) was used. Cyanobacterium was grown on Fitzgerald medium with the modification by Zehnder and Gorham [8] in Erlenmeyer flask at 22°C ± 2°C and illumination of 2500 lux until the stationary growth phase. The concentration of chlorophyll (Chl) in cyanobacterial cells was determined by differential fluorometry (Fluorometer FL300 3M, Russia) [9]. The binary composition was prepared by mixing the bacterial suspensions consisting of nodule bacteria (1 × 10^9^ cells/mL) and cyanobacteria (Chl, mg/L = 1506,6 ± 13,4, Δ*F* = 0,088) in the ratio 1 : 1. In parallel, the viability of cyanobacterial cells was determined by the difference of fluorescence intensity (Δ*F*) before and after the addition of simazine, the inhibitor of cells photosynthetic electron transport [10, 11].

Investigations were carried out in the model experiments in a growth area of Institute of Plant Physiology and Genetics with natural light and humidity of the substrate 60% of full capacity. Plastic containers with 10 kg of sand were used in experiments. 12 alfalfa plants were grown in each container. Containers were preliminarily sterilized with 20% solution of H_2_O_2_. Washed river sand with the mineral nutrient mixture of Gelrigel [12] containing the “start” of nitrogen (177 mg of Ca(NO_3_)_2_  × 4H_2_O per 1 kg of sand) was used as a substrate. This amount of nitrogen represents one-quarter of the normal nitrogen supply. Before sowing the seeds were sterilized with concentrated sulfuric acid for 5 minutes, and then they were washed in running water for 1 h. The treatment of seeds by microorganism compositions was continuing during 1 h.

The controls in the experiments were the samples of seeds treated by monoculture of T17* S. meliloti* or only by* N.* PTV. We used samples of alfalfa seeds moisturized with tap water as an additional “absolute” control. Experiments were performed in seven replications. Plants for analysis were selected in phases of stem (32nd day of emergence), budding (40th day), and flowering (50th day).

### 2.2. Measurements of Nitrogen Activity, Pigments Content, and Efficiency of Photosynthesis

Nitrogen-fixing (nitrogenase) activity was determined by the level of activity of root nodules by acetylene method and expressed as micromoles of ethylene formed by nodules per plant for 1 h [13]. The gas mixture was analyzed by gas chromatography of Agilent Technologies 6855 Network GC System (USA). The measurements were performed in five replications.

The content of the photosynthetic pigments in leaves of alfalfa plants was determined by the Wellburn method [14]. Pigments were extracted with dimethyl sulfoxide (0.1 g vegetable material was treated in 10 mL DMSO) of leaf cut for 3 h at +67°C until complete extraction. The absorbance of the solution was measured by spectrophotometer Smart Spec Plus (BioRad, USA) at 665 and 649 nm in a 1 cm cuvette. Leaves were collected from the middle tiers of the five randomized plants of the same version. Measurements were performed in triplicate.

The net assimilation rate of shoots was determined in controlled environment with installation built on base of the photoacoustic infrared gas analyzer GIAM-5 M (Russia), which was connected by differential circuit. Container with plants was placed in sealed plexiglass chamber of 50 liters through which air was blown at rate of 15 L/min. At the outlet of chamber 1 L/min of air was taken to the gas analyzer, and the remaining air was discharged into atmosphere. The chamber was irradiated with light by the lamp CG-2000 through a water filter. The illumination on the substrate level was 250 W/m^2^; temperature was 25°  ±  2°C. After the adaptation of plants to the conditions of measurement (30–40 min after closing the chamber), the rate of absorption of CO_2_ by plants was recorded (it is an apparent photosynthesis). After this, shoots of plants were cut at the substrate level and respiration of soil with roots were measured. Net assimilation rate was calculated as sum of apparent photosynthesis and respiration. Calculations of gas exchange parameters were performed according to the standard procedure [15].

The protein content was determined in leaves of alfalfa plants in the budding stage by Lowry method [16]. Qualitative and quantitative composition of amino acids was determined by liquid-ion exchange column chromatography with the use of automatic analyzer T339 (Czech) on the basis of ninhydrin detection method [17].

### 2.3. Plant Stress Resistance Determination

In order to study the effect of mono- and binary inoculation on plant resistance, the basic parameters of the stress state of alfalfa were determined. Plants in the budding stage were treated with herbicide diquat (100 pmol), which was used as a stress factor. Sampling was carried out after 30 minutes, 60 minutes, and 24 hours of diquat action on plants. Specific changes in the composition of the components of a lipid-pigment complex and antioxidant system were studied in photosynthetic tissues of alfalfa.

Intensity of lipid peroxidation (LPO) was evaluated by the number of end-products of lipid oxidation based on the reaction with 2-thiobarbituric acid (TBA) [18]. The activity of antioxidant systems is determined by the activity of superoxide dismutase (SOD) [19].

A statistical analysis of the experimental data was performed by standard methods, involving a package of special statistical functions of Microsoft Excel. Probability of differences between the variants was assessed by *t*-test and a significance level of *P* < 0,05.

### 2.4. Scanning Electron Microscopy (SEM)

Cyanobacterial samples were fixed as described above and dehydrated through an ethanol series, with an overnight exposure in absolute acetone followed by critical-point drying in a Dryer HCP-2 (Hitachi, Japan), coated with Au-Pd alloy in an IB-3 Ion Coater (Eiko, Japan) and examined with a JSM-6380LA scanning electron microscope (JEOL, Japan).

### 2.5. DNA Isolation and PCR Amplification

For molecular typing cyanobacterial genomic DNA was isolated according to [20] and synthetic oligonucleotides (“Synthol,” Moscow, Russia) have been used as cyanobacterial primers for 16S–23S rRNA PCR, according to [21]. As a second molecular marker the* nifH* gene has been used with corresponding PCR primers [22]. PCR for 16S–23S rRNA gene cluster was carried out on a Tercik DNA amplifier (DNA Technology, Russia) by using DreamTaq PCR Master Mix (Fermentas, EU), under the following conditions: 1 cycle at 94°C for 10 min, 25 cycles at 94°C for 45 sec, 54°C for 45 sec, 68°C for 2 min, 1 cycle at 68°C for 7 min, and a final soak step at 4°C. PCR for partial* nifH* gene was performed under the following conditions: 1 cycle at 94°C for 4 min, 25 cycles at 94°C for 30 sec, 54°C for 30 sec, 68°C for 30 sec, 1 cycle at 68°C for 7 min, and a final soak step at 4°C. PCR products were resolved in 1.5% agarose gel containing ethidium bromide at 5 microgram mL^−1^.

### 2.6. Cloning and Sequencing of PCR Products

DNA fragments obtained during PCR were cloned with CloneJet PCR Cloning Kit # K1231 (Fermentas, EU). Transformation of competent XL-1 cells of* Escherichia coli* and plasmid purification were performed according to [23]. DNA sequencing was performed with ABI PRISM BigDye Terminator version 3.1 at the Applied Biosystems 3730 DNA Analyzer (Center for Collective Use “Genome”). Sequences were edited and assembled with Bioedit (Invitrogen, Carlsbad, CA). The full nucleotide sequence of the rRNA gene cluster of cyanobacterium* Nostoc* PTV and a part of the* nifH* gene were accomplished and deposited to GenBank under accession numbers JQ259185.1 and JQ259186.1.

### 2.7. Phylogenetic Analysis

Search of the nucleotide sequences in the database GenBank, homologous to the sequenced genes of studied species of cyanobacteria, was performed using BLAST (https://blast.ncbi.nlm.nih.gov/Blast.cgi?PROGRAM=blastn&PAGE_TYPE=BlastSearch&LINK_LOC=blasthome) with the option: the least degree of similarity (Minimum Identity). The sequences of selected species were aligned using the algorithm Muscle (MEGA 6.0) [24]. Phylogenetic reconstructions were performed using Bayesian inference (MrBayes version 3.1.2) [25] with the preselection of an adequate model of nucleotide substitutions (MEGA 6.0).

### 2.8. Mating and Conjugal Transfer of Plasmid DNA

Transformations of* Nostoc* PTV through triparental conjugations followed published protocols [26] with minor modifications. Standard bacterial mating involved the cyanobacterial strain* Nostoc* PTV and* E. coli* strains (DH10B) that harbored the following three plasmids: (i) the conjugal plasmid pRL443 [27], (ii) the “helper” plasmid pRL623, [27], and (iii) the cargo plasmid pRL692 that carries the mobile element Tn5-692 [28].* E. coli* strains were grown in 3 mL LB with the appropriate antibiotic(s) and incubated at 37°C overnight. Cells of* E. coli* were diluted 1 : 20 and were grown for 1.5–2 h at 37°C. Then* E. coli* cells were harvested from 1 mL of each* E. coli* culture by centrifugation and resuspended in 1 mL fresh LB. This step was repeated twice to wash the cells. After the third centrifugation, the cells were resuspended in 200 mL BG-11. Five milliliters of a growing* Nostoc* PTV culture was harvested by centrifugation at low speed (4000 g) and resuspended in 1 mL BG-11. Then the filaments were fragmented in a water bath sonicator for 2 to 5 min so that more than half of the filaments were shorter than 5 cells. The cyanobacterial cells were collected by centrifugation for 2 min and resuspended in 1 mL BG-11. The cargo strain, the conjugal strain (for triparental mating), and* Nostoc* PTV were combined, pelleted by centrifugation, and finally resuspended in 200 mL BG-11. The conjugation mixture was incubated for about 1 h in low light at 28°C. Then the cells were spread on sterile nitrocellulose filters laid on BG11+ 5% (vol/vol) LB agar plates (mating plates). The mating plates were incubated without antibiotic selection for 18 to 24 h in low light at 28°C, and then the filters were transferred to BG-11 for 24 h and then to BG-11 agar with 10 *μ*g/mL Spectinomycin (Sp10) and 2 *μ*g/mL Streptomycin (Sm2). After incubation for 8 to 12 days, isolated antibiotic-resistant transconjugant colonies were patched on fresh selective BG-11 plates.

## 3. Results and Discussion

### 3.1. Effect of Microbial Inoculation on Plant Growth and Productivity

Earlier in the laboratory study of pure cultures of* N*. PTV and* S. meliloti* [29], we found stimulation of cell growth area of nodule bacteria around the colonies of cyanobacterium* N.* PTV on the surface of the agar medium. Our results are consistent with the literature data, since it is known that cyanobacteria are producers of a wide range of biologically active substances, which include a group of growth-stimulating compounds, analogues of phytohormone [30].

In our previous study we have tested different associations of nitrogen-fixing microorganisms in the rhizosphere of alfalfa [29]. The most effective bacterial consortium included cyanobacterium* Nostoc* PTV and Tn5-mutants of nodule bacteria* S. meliloti.* Usage of the optimal proportions of components in the inoculation mixtures promotes the absence of antagonism between microorganisms and provides the stimulating effect of these consortia on various physiological and biochemical features of alfalfa plants.

**Figure 1:**
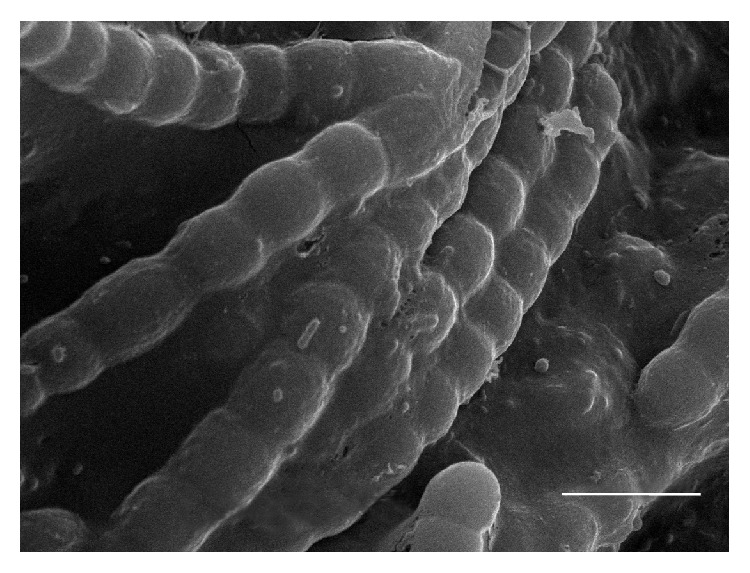
A SEM image of the* N.* PTV cells. Scale bar: 5 *μ*m.

In this study the possibility of the formation of artificial stable microbial consortium based on nitrogen-fixing cyanobacterium* N.* PTV (Figure 1) and one Tn5-mutant of nodule bacteria* S. meliloti* T17 was investigated. In pot experiments it was revealed that inoculation of alfalfa by binary mixture of* S. meliloti* T17 +* N*. PTV has a stimulating effect on the growth of the vegetative mass of plants (Table 1). The increase of above-ground plant mass after application of the consortium* S. meliloti* T17 +* N.* PTV in the phase of stem was 15.4% compared with rhizobial T17 monoinoculation and 21% compared with* N.* PTV monoinoculation, respectively. The growth of above-ground alfalfa plant mass was 13.8% and 10.4%, after application of the* S. meliloti* T17 +* N.* PTV consortium in the budding stage, and 5% and 13% in the beginning of flowering, in comparison with monoinoculations, correspondingly. It is known that cells of nitrogen-fixing cyanobacteria produce polypeptides, amino acids, polysaccharides, and vitamins. Due to this diverse biochemical activity in the mucous environment of cyanobacterial cells favorable conditions for growth and reproduction of other microorganisms were created. Perhaps, it could promote a more active cell proliferation of nodule bacteria T17 associated with cyanobacteria in the root zone of alfalfa and contribute to formation of efficient* Rhizobium*-legume symbiosis.

Rhizogenesis was positively affected in plants, the seeds of which were treated with suspensions of microorganisms (Table 1). The largest increase of the plant root mass was detected after using the binary inoculation (*S. meliloti* T17 +* N*. PTV). Thus, in the phase of stem the mass of roots increased by 46.6% and 22.2%, in the budding stage by 49.2% and 13%, and in the early phase of flowering by 13.9% and 39.8%, compared with plants treated only by* S. meliloti* T17 or by only* N.* PTV, correspondingly.

Effective collaboration between all partners of symbiosis provides the activation of several metabolic processes and, above all, the fixation of atmospheric nitrogen. As a result of improved plant nutrition, their productivity increased and the quality of bioproducts improved.

**Figure 2:**
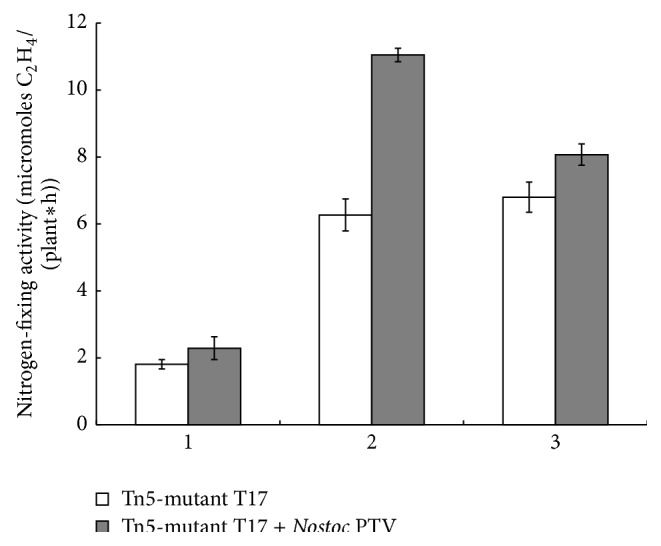
Dynamics of NFA of nodules of alfalfa plants inoculated by mono- and binary suspensions of microorganisms (micromoles of ethylene formed by nodules per plant per 1 h). 1: phase of stooling, 2: phase of budding, and 3: phase of flowering, *P* ≤ 0,05.

In the phase of stem when the process of nitrogen fixation was still inactive, differences in NFA of nodules of alfalfa plants inoculated by mono- or binary bacterial complexes were not significant (Figure 2). However, in the budding stage and in the early flowering stage a nitrogen fixation in root nodules of plants infected with a mixture of* S. meliloti* T17 +* N*. PTV was more intensive. Positive regulatory role of cyanobacterium* N*. PTV is obvious according to the results presented in Table 2. Only in case of binary inoculation, plants demonstrate an increase in number and in weight of formed nodules (Table 2). Thus, the application of this microbial consortium provided increased NFA in nodules of alfalfa at the budding stage and maintained its relatively high level at the beginning of the early flowering stage (Figure 2). Therefore, this data proves that* N*. PTV has a stimulating effect on the functioning of root nodule bacteria* S. meliloti* T17.

It is known that the use of active strains of root nodule bacteria and their associations with other microorganisms affect the formation and functioning of the photosynthetic complexes through the nitrogen status of a host plant. Presence of nitrogen available to plants determines the efficiency of symbiotic systems.

**Figure 3:**
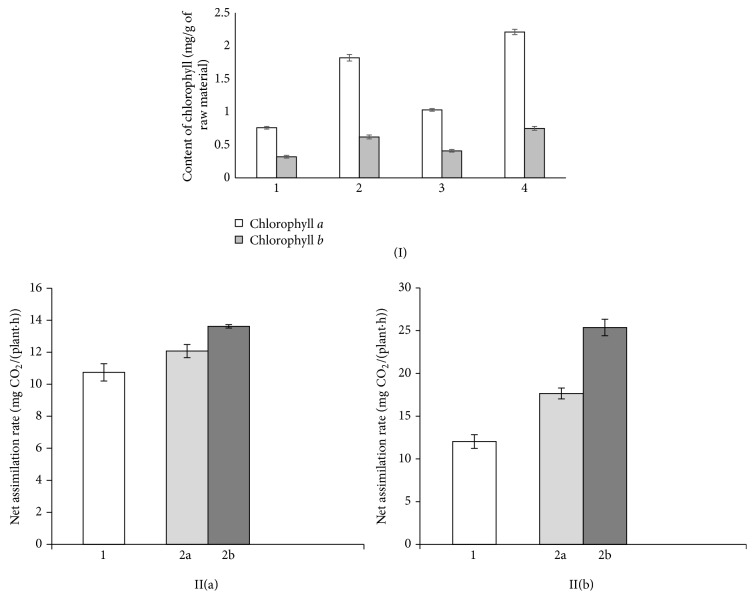
(I): content of chlorophyll (mg/g of raw material) in leaves of alfalfa inoculated by mono- and binary suspensions of microorganisms* S. meliloti* Т17 and* N.* PTV. 1: control (without inoculation); 2: inoculation by *N*. PTV; 3: inoculation by* S. meliloti* Т17; 4: inoculation by consortium of* S. meliloti* Т17 +* N.* PTV. II: net assimilation rate (mg СО_2_/( plant·hour)) of alfalfa inoculated by mono- and binary suspensions of microorganisms* S. meliloti* Т17 and* N.* PTV. 1: inoculation by* N*. PTV; 2a: inoculation by* S. meliloti* Т17; 2b: inoculation by consortium of* S. meliloti* Т17 +* N.* PTV (II(a): phase of budding and II(b): phase of flowering).

Mono- and binary suspensions inoculations of seeds showed positive dynamics of accumulation of photosynthetic pigments in the leaves of alfalfa compared with the absolute control (Figure 3). The most significant differences were observed in plants whose seeds had been inoculated with nitrogen-fixing consortium of microorganisms (chlorophyll* a* and chlorophyll* b* increased by 114.6 and 82.9%) compared with the corresponding option of treatment only by strain T17. It is known that the content of chlorophyll in the leaves is directly proportional to the intensity of nitrogen fixation and depends on symbiotic properties of root nodule bacteria [31–33]. Increasing the number of pigments in the leaves of alfalfa inoculated with binary bacterial suspension indicates the ability of* N.* PTV to enhance the functional activity of rhizobia, which are directly interfaced with the intensity of nitrogen fixation.

Available forms of nitrogen, such as mineral and symbiotrophic, positively affect not only the formation of high grade, but also functional state of the plant photosynthetic apparatus. The net assimilation rate also demonstrates the effectiveness of the binary composition* S. meliloti* T17 +* N*. PTV. In particular, in the budding stage of these plants the net assimilation rate exceeded 12.7%, while in the phase of early flowering it increased by 43.7% of the corresponding rate during inoculation only by T17 (Figure 3).

The net assimilation rate of plant leaves typically is closely correlated with the content of nitrogen, and nitrogen is presented mainly in amino acids and proteins. Rubisco, the major cell photosynthetic enzyme of CO_2_ assimilation, represents more than half of the soluble cell proteins in leaf. Obviously, the intensification of NFA in the binary composition was the main reason for the increase of plant assimilation rate. However, it is possible that more active symbiotic apparatus, which is formed on the roots of plants through binary inoculation, enhanced “request” on assimilates by the root system, thereby stimulating the photosynthetic activity of plant leaves. There is a gradient of transport forms of carbon, particularly sucrose, between roots and leaves in the conduction system and it accelerates the outflow of carbon from the leaves. This, in turn, eliminates restrictions by photosynthesis products imposed on the feedback principle and further accelerates photosynthetic carbon assimilation. Thus, the efficient operation of the symbiotic apparatus in inoculated plants greatly stimulated the accumulation of photosynthetic pigments and increased the net assimilation rate. The accumulation of organic matter contributes to the formation of the plant biomass, because the basis of the biological productivity of the plant organism, including those capable of symbiotic nitrogen fixation, is photosynthetic carbon assimilation [31].

In consequence of artificial inoculation of alfalfa seeds by consortium of nitrogen-fixing microorganisms* S. meliloti* T17 +* N*. PTV the yield of green mass of plants increased by 17.9% and the protein content in the leaves increased by 12.0% compared to monoinoculation by strain T17 (Table 3). This is an evidence of the effective interaction of test organisms in the cyano-*Rhizobium* associations and their positive impact on the growth and physiological characteristics of alfalfa plants (Table 3).

**Figure 4:**
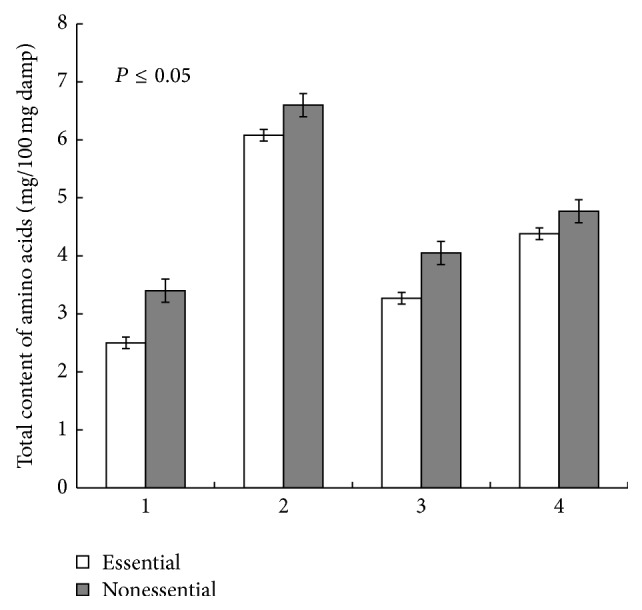
Total content of essential and nonessential amino acids in leaves of alfalfa grown after mono- and binary inoculation by cyanorhizobial compositions of microorganisms: 1: control (without inoculation), 2: monoinoculation of alfalfa seeds by cyanobacterium* N.* PTV, 3: inoculation of alfalfa seeds by Tn5-mutant strain of* S. meliloti* T17, and 4: binary inoculation of alfalfa seeds by Tn5-mutant strain of* S. meliloti* T17 +* N*. PTV.

The amino acid composition is the main criterion of the biological value of proteins. An index of a total amino acid composition of vegetative mass of the experimental inoculated variants of alfalfa plants increased in comparison to the control (without bacterial inoculation). The maximum quantity of lysine, the most essential and deficient amino acid in humans and animals, was recorded in leaves of alfalfa (Table 4). As a result of using binary inoculation a total amino acid composition increased by 25.1%, compared with the case of inoculation only by T17. In particular, a quantity of essential amino acids increased by 33.9%, and a quantity of nonessential amino acids increased by 17.7% (Figure 4). At the same time, an increase of the content of methionine, histidine, arginine, and tyrosine was observed, which are present in small quantities in plant leaves, and this is one of the factors limiting the rate of biosynthesis of proteins, especially in generative organs. The results are a direct proof of the positive impact of cyanobacterial inoculation on the quality of agricultural products.

### 3.2. Stress Response of Plants Inoculated with Microbial Consortium

**Figure 5:**
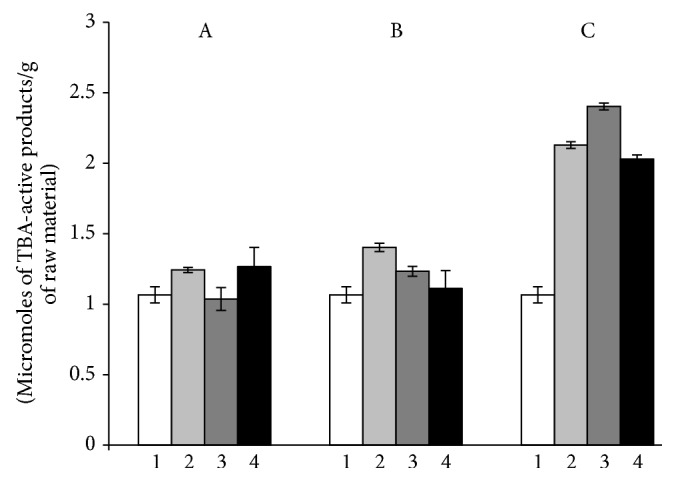
Content of TBA-active products in alfalfa leaves after herbicide diquat treatment: А: for 30 min, B: for 60 min, and C: for 24 h. 1: control (without inoculation and herbicide diquat treatment); 2: control (without inoculation, with herbicide diquat treatment); 3: inoculation by Tn5-mutant of* S. meliloti* Т17, with herbicide diquat treatment; 4: inoculation by Tn5-mutant of* S. meliloti* Т17+* N*. PTV, with herbicide diquat treatment.

**Figure 6:**
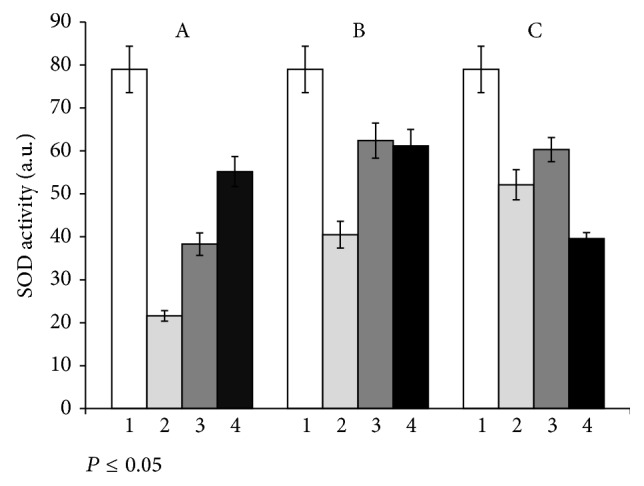
SOD activity in alfalfa leaves after herbicide diquat treatment: А: in 30 min, B: in 60 min, and C: in 24 h. 1: control (without inoculation and herbicide diquat treatment); 2: control (without inoculation, with herbicide diquat treatment); 3: inoculation by Tn5-mutant of* S. meliloti* Т17, with herbicide diquat treatment; 4: inoculation by Т17 +* N*. PTV, with herbicide diquat treatment.

It is known that the plant productivity rate and resistance index are inversely dependent values. A positive effect of the cyanobacterial consortium T17 +* N.* PTV on the productivity of alfalfa is shown in our study. It was logical to study the effect of the binary inoculation on plant resistance to the adverse effects of certain environmental factors. It is an extremely important issue. We have used herbicide diquat as a model stress factor. For a short duration (30 minutes) of diquat treatment the content of TBA-reactive products in photosynthetic tissues of plants that were inoculated with the strain T17 was reduced by 18% compared to plants without inoculation (control 2). In the case when plants were inoculated with the consortium* S. meliloti* T17 +* N*. PTV the content of TBA-reactive products remained at the level of control. In the experiments with more prolonged action of the stress factor (60 min) the content of TBA-reactive products in plants inoculated only by the strain T17 decreased by 16.9% and in the case of binary inoculation the content of TBA-reactive products decreased by 25%. It should be noted that after 24 h of plants exposure with diquat, regardless of the inoculation agent used, reducing the amount of TBA-active products in photosynthetic tissues was not observed compared with the control. At the same time, in the experiment with the use of the consortium of microorganisms a difference (15.4%) with the inoculation only by strain T17 was marked (Figure 5). At short-term action of diquat (30 min) a rate of SOD activity in photosynthetic tissues of plants inoculated with* S. meliloti* T17 +* N*. PTV was 2.5 times higher than in controls and by 42.5% in plants, inoculated by strain T17. After herbicide treatment during 60 minutes a significant altering of SOD activity in inoculated plants (irrespective of whether a mono- or binary inoculation) was not observed. However, in comparison to the control, this difference was significant; the enzyme activity was increased by 54%. Under long-term stress (24 h) in plants inoculated with strain T17, SOD activity remained at the same level as for short-term exposure. In plants, the seeds were treated with consortium* S. meliloti* T17 +* N*. PTV, for the same conditions; this index decreased by 23.4% compared with the control and 34.4%, in comparison with the plants inoculated by strain T17 (Figure 6). Thus, the plants inoculated with algae-rhizobial composition proved to be more resistant to oxidative stress. It is possible due to the increased level of NFA of their symbiotic system and thus the increase in the number of available forms of nitrogen for alfalfa plants and the possible participation of NO in the defense reactions. In the literature, there are two hypotheses about the mechanisms of NO action under conditions of stress. First, NO may act as an antioxidant, directly linking to ROS, thereby protecting cells from damaging their actions [34]. Secondly, NO can act as a signaling molecule that triggers a cascade of reactions that lead to the expression of specific genes [35]. In their chemical and physical properties small molecule, rapid metabolism, lack of charging, and high diffusion coefficient of NO are well suited for the role of intracellular signaling mediator of plant stress responses.

Thus, the inoculation of alfalfa seeds by a consortium of nitrogen-fixing microorganisms* S. meliloti* T17 +* N*. PTV increased the nitrogenase activity of root nodules, increased the net assimilation rate, and increased productivity and product quality, and also the stability of alfalfa plants under the influence of oxidative stress induced by herbicides.

### 3.3. Molecular Typing and Phylogenetic Analysis of Cyanobacterium* Nostoc* PTV

One of the main goals of this study was the molecular typing and phylogenetic analysis of a new cyanobacterial isolate* N*. PTV originated from the Institute of Hydrobiology of Academy of Science of Ukraine. As it was shown above, this cyanobacterium is effective for soil algalization. As a component of algae-rhizobium compositions, this cyanobacterium stimulates germinative energy, growth, and productivity of legumes.

To identify and to determine the phylogenetic positions of the new cyanobacterial isolate* N*. PTV we used a partial sequence of the* nifH* gene (342 bp), encoding nitrogenase reductase, and 16S ribosomal RNA gene cluster (1765 bp) as molecular markers. Comparison of the* nifH* gene sequence and rRNA gene cluster sequence of cyanobacterium* N.* PTV with all the sequences present at the GenBank by using the program Blast (https://blast.ncbi.nlm.nih.gov/Blast.cgi?PROGRAM=blastn&PAGE_TYPE=BlastSearch&LINK_LOC=blasthome) shows that this strain has no full similarity with any of early investigated organisms.

**Figure 7:**
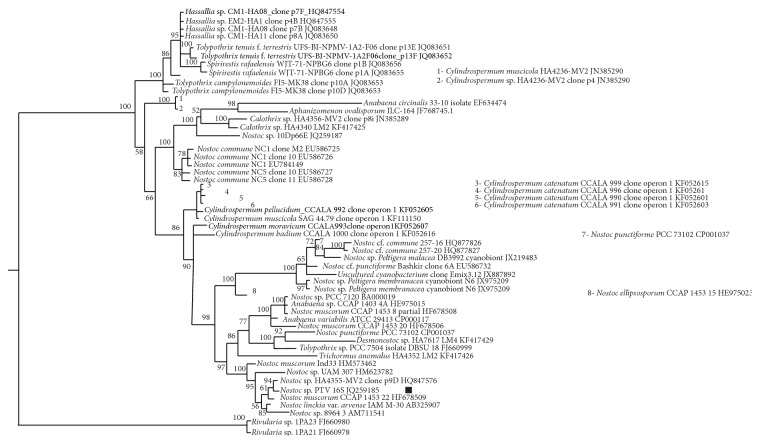
Phylogenetic relationships of* Nostoc* sp. PTV (designated by black square) inferred under the posterior probability criterion (MrBayes) from the gene for 16S rRNA, partial sequence information. Numbers at the nodes indicate the Bayesian statistical support values (posterior probabilities multiplied by 100); only values higher than 50% are given. The scale bar indicates the number of substitutions per nucleotide position.

**Figure 8:**
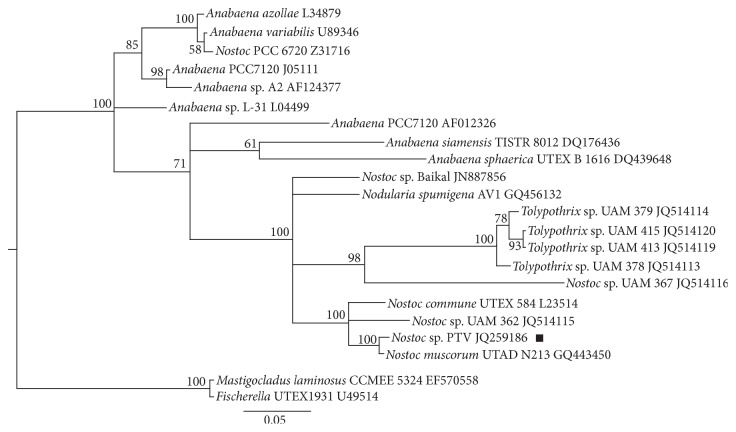
Phylogenetic relationships of* Nostoc* sp. PTV (designated by black square) inferred under the posterior probability criterion (MrBayes) from the gene for* nifH*, partial sequence information. Numbers at the nodes indicate the Bayesian statistical support values (posterior probabilities multiplied by 100); only values higher than 50% are given. The scale bar indicates the number of substitutions per nucleotide position.

Comparison of rRNA gene cluster sequence (1765 bp) of the cyanobacterium* Nostoc* sp. PTV revealed that this cyanobacterium shows the highest similarity with several strains of* N. muscorum* and* N. commune* (Table 5). In general, support for branching in the tree, based on a fragment of 16S rRNA gene sequence (Figure 7), is worse in comparison with the reconstruction of the phylogeny of cyanobacteria based on the sequence of the gene* nifH* (Figure 8).

The group of* Nostoc* strains and species, which includes* Nostoc* PTV, forms a cluster with the minimum of allowable support, 0.95. Hierarchy of strains (HA 4355-MV2, PTV, and 8964) and* Nostoc* species (*N. muscorum* and* N. linckia*) cannot be evaluated because of the low topological support of this site of the tree: credibility values range from 0.56 to 0.94. It is difficult to discuss a relation of* Nostoc *PTV strain to strain HA 4355-MV2, due to the very low values of the other branches in the cluster, and very scarce information about the HA-MV2 4355 strain clearly does not help to solve the problem of their possible relationship.

*N. muscorum* is discussed below.* N. linckia* is also a freshwater strain and could be isolated in some terrestrial niches. Interestingly, the* Nostoc* strain UAM 307 is quite clearly differentiated from the other representatives of Nostocaceae (0.95). Being sufficiently close to strain of* Nostoc* PTV, this cyanobacterium has some significant features that provided the separation of this strain into the other branch of the common with* Nostoc* PTV cluster.

Even more interesting detail is that the last significant branch (credibility value is equal to unity) of the cluster is formed by the strain of* N. muscorum* Ind33, which is significantly aside not only from the desired strain of PTV, but also from the different strains of the same species (CCAP 1453-22). Thus, the strain of* Nostoc* PTV has teamed up with members of their own genus.

Outgroup of this dendrogram is represented by two strains of* Rivularia* (this kind of cyanobacteria forms heteropolar threads; their trichomes are densely agglomerated, covered with a total mucus). The genus is represented only by the species often living on calcareous substrates, but there are rare epilithic and epiphytic species.

Comparison of* nifH* sequence of cyanobacterium PTV revealed that this strain shows the highest similarity with several strains of* Nostoc* (Table 6). The closest relative is* Nostoc muscorum* UTAD N213, purified from rice paddy in Mondego River Basin (Portugal). Phylogenetic analysis (Figure 8) revealed that the cyanobacterium PTV forms a minicluster with* Nostoc muscorum* UTAD N213.* N. muscorum* is a free-living filamentous cyanobacterium, which inhabits both terrestrial and freshwater aquatic environments. They are phototrophic organisms performing photosynthesis and also fixing atmospheric nitrogen [36].

*N. muscorum* is the most common type of* Nostoc* in terrestrial ecosystems and is widely spread, due to the adaptability to many adverse conditions. It forms a symbiotic relationship with many types of terrestrial plants and fungi.

It is known that* N. muscorum* has great effect on soil biology and productivity which makes it an attractive soil inoculant. This cyanobacterium is able to obtain carbon and nitrogen from the air and has an advantage over heterotrophic soil inoculants, which are usually limited by carbon [37].

It also benefits plants and other soil bacteria by increasing soil organic matter in the form of carbohydrates and provides biological organic nitrogen that can be assimilated by plants [38].* N. muscorum* helps to create the environment conditions to further colonization and growth by plants and other microorganisms [39].

Inoculation of the* N. muscorum* isolates caused a significant effect on growth of wheat and maize plants. Cyanobacterial inoculation positively affected pigment content, increased plant shoot and root dry weight, and increased leaf area [40].

In general, the topology of the dendrogram (Figure 8) has a good support; the main branch nodes are characterized by high credibility values.

*Nostoc* PTV is in the same cluster with* Nostoc* sp. UAM-362 and* Nostoc commune*.* Nostoc* sp. UAM-362 was isolated from the rock surface of calcareous river with brackish water in Spain: Muga, Girona (Northeast Spain).* N. commune* is a colonial species of cyanobacterium. As well as* N. punctiforme, N. commune *is able to survive in extreme conditions such as polar regions and arid areas.

Three more clusters are presented as the parts of the large one: two single clusters, represented by* Nostoc* sp. Baikal (nitrogen-fixing cyanobacteria from Lake Baikal) and by* Nodularia spumigena* from family Nostocaceae.* Nodularia* occurs mainly in brackish or saline waters.* Nodularia* cells occasionally can form heavy algal blooms. Some strains produce a toxin (nodularin), which is harmful to human health [41].

The third cluster is formed by four strains of* Tolypothrix* and by one strain of* Nostoc* sp. UAM-367 (isolated from rock surface of calcareous river with brackish water in Spain: Muga, Girona). Cyanobacteria* Tolypothrix* grow in unpolluted waters; several species are found in swamps, known aerophilic species growing on the bark of trees, in the wet sands, on wet rocks, and so forth.

Two species, and one strain (PCC 7120) of* Anabaena*, representing a family of filamentous cyanobacteria Nostocaceae, belong to this large cluster with a poor resolution (a credibility value of branching is 0.71). These cyanobacteria exist in the form of plankton; some species are symbionts of plants.* Anabaena* is one of the four genera of cyanobacteria that produce neurotoxins.* Anabaena* is a model for the study of cell differentiation and differential gene expression during nitrogen fixation [42].* A. siamensis* and* A. sphaerica* are freshwater species. This mega cluster consisting of described species of cyanobacteria is well differentiated from the other two clusters: a single one represented by strain* Anabaena* L-31 (freshwater cyanobacterium) and a poorly differentiated cluster, which consists of two strains of* Anabaena* (A2 and PCC 7120) and two well-differentiated species of* Anabaena, A. azollae* and* A. variabilis*.* A. azollae* forms symbiosis with water fern* Azolla*.

Outgroup for the described species of* Nostoc* and* Anabaena* is represented by *Mastigocladus laminosus* and by member of the genus* Fischerella* (strain UTEX 1931). The first organism is a typical representative of the genus* Mastigocladus*.* Fischerella* represents another squad, Stigonematales. Both types of reference are truly branching filamentous forms of thermophilic cyanobacteria.

### 3.4. Gene Transfer into* Nostoc* PTV Cells

*Nostoc* PTV cells were tested for their ability to conjugational DNA transfer. As a result of plasmid pRL692 transfer into cyanobacterial cells several hundred transconjugant colonies were selected on selective plates that contained solid BG-11 medium and antibiotics (Sp10 and Sm2). On control plates antibiotic-resistant colonies were absent (data not shown). In future experiments we plan to use this experimental approach for transposon mutagenesis of* Nostoc* PTV and selection of the new mutants with interesting characteristics.

## 4. Conclusions

The use of microbial consortium newly identified* Nostoc* PTV strain together with* Sinorhizobium meliloti* T17 was more efficient than the use of the single-rhizobium strain for alfalfa plant inoculation. This treatment provides an intensification of the processes of nitrogen fixation and photosynthesis and stimulates growth of above-ground plant mass and rhizogenesis and leads to increased productivity of* Medicago sativa* L. and improved amino acid composition of plant leaves. Phylogenetic analysis by using two different molecular markers showed that this new cyanobacterium belongs to a cluster of the genus* Nostoc*, with the closest relative of* Nostoc muscorum*. Gene transfer of transposon bearing plasmid DNA has been shown for cyanobacterium* Nostoc* PTV. It makes this strain very attractive model for future genetic and physiological experiments and biotechnological applications.

## Figures and Tables

**Table 1:** Dynamics of accumulation of vegetative mass of alfalfa inoculated by mono- and binary suspensions of diazotrophic microorganisms.

Inoculants	Phase of plant development					
	Stooling		Budding		Flowering	
	Above-ground mass (g/plant)	Mass of roots (g/plant)	Above-ground mass (g/plant)	Mass of roots (g/plant)	Above-ground mass (g/plant)	Mass of roots (g/plant)
Without inoculation (control)	0,42 ± 0,02	0,12 ± 0,01	1,17 ± 0,06	1,12 ± 0,1	1,25 ± 0,02	2,25 ± 0,16
*N. *PTV	0,62 ± 0,02	0,18 ± 0,01	1,64 ± 0,09	1,79 ± 0,09	1,70 ± 0,07	2,11 ± 0,15
*S. meliloti* Т17	0,65 ± 0,04	0,15 ± 0,01	1,59 ± 0,13	1,22 ± 0,11	1,83 ± 0,04	2,59 ± 0,24
*S. meliloti* Т17 + *N. *PTV	0,75 ± 0,08	0,22 ± 0,01	1,81 ± 0,18	1,82 ± 0,10	1,92 ± 0,03	2,95 ± 0,29

*P* ≤ 0,05.

**Table 2:** Number and mass of root nodules on alfalfa plants inoculated by mono- and binary suspensions of microorganisms.

Inoculants	Phase of plant development					
	Stooling		Budding		Flowering	
	Number of root nodules (pcs/plant)	Mass of root nodules (g/plant)	Number of root nodules (pcs/plant)	Mass of root nodules (g/plant)	Number of root nodules (pcs/plant)	Mass of root nodules (g/plant)
Without inoculation (control)	0	0	0	0	0	0
*N. *PTV	0	0	0	0	0	0
*S. meliloti* Т17	12,0 ± 1,0	0,010 ± 0,00	30,0 ± 8,5	0,115 ± 0,002	45,0 ± 0,5	0,135 ± 0,02
*S. meliloti* Т17 + *N. *PTV	14,0 ± 0,6	0,017 ± 0,04	57,0 ± 8,0	0,130 ± 0,001	70,0 ± 7,5	0,160 ± 0,02

*Note*. 15 plants of each variant of the experiment were analyzed for determination the average number of nodules on the roots of one plant.

*P* ≤ 0,05.

**Table 3:** Productivity and protein content in leaves of alfalfa, inoculated by mono- and binary suspensions of microorganisms.

Inoculants	Harvest of green mass of plant, g/vessel				Protein content in the leaves	
	I mowing	II mowing	Total harvest	% to monoinoculation by rhizobium		% to monoinoculation by rhizobium
Control	17,68 ± 0,51	19,70 ± 0,64	37,38		13,2	
*N*. PTV	21,40 ± 0,52	24,22 ± 0,76	45,62		14,6	
*S. meliloti* Т17	21,81 ± 0,48	25,17 ± 0,30	46,98		18,32	
*S. meliloti* Т17 + *N. *PTV	26,26 ± 0,37^∗^	29,15 ± 0,17^∗^	55,41	**117,9**	20,52	**112,0**

**Table 4:** Amino acid content in leaves of alfalfa, inoculated by mono- and binary suspensions of microorganisms.

Amino acid	Content of amino acids (mg/100 mg DW)			
	Control	*N*. *punctiforme *	Т17	Т17 + *N. punctiforme *
Gamma-aminobutyric acid	0,065	0,088	0,085	0,123
Lysine	**0,386**	**0,802**	**0,459**	**0,610**
Histidine	0,094	0,301	0,171	0,232
Arginine	0,297	0,905	0,439	0,585
Asparagine	0,675	0,882	0,748	0,779
Threonine	0,278	0,623	0,383	0,496
Serine	0,321	0,698	0,413	0,533
Glutamic acid	0,876	1,931	1,083	1,395
Proline	0,376	0,610	0,419	0,548
Glycine	0,423	0,790	0,470	0,537
Alanine	0,479	0,895	0,581	0,644
Cysteine	0,054	0,262	0,057	0,079
Valine	0,245	0,651	0,301	0,423
Methionine	0,111	0,298	0,149	0,200
Isoleucine	0,175	0,439	0,233	0,273
Leucine	0,523	1,234	0,685	0,938
Tyrosine	0,201	0,534	0,285	0,253
Phenylalanine	0,398	0,835	0,453	0,626
Total	**5,977**	**12,779**	**7,417**	**9,276**

**Table 5:** BLAST results obtained by querying the 16S–23S rRNA gene cluster of *Nostoc *sp. PTV with GenBank and geographical and ecological origins of the hits.

Closest GenBank relative	GenBank access number	Query coverage, %	Score, %	Identity, %	*E* value	Origin of the strain and reference
*Nostoc* sp. HA4355-MV2 clone p9D	HQ847576	98	2872	97	0.0	Maniniholo Cave wall, near Haena USA: Kauai, Hawaii

*Nostoc muscorum* CCAP 1453/22	HF678509	98	2531	93	0.0	Scottish Association for Marine Science, Molecular and Microbial Biology, Dunstaffnage Marine Laboratory, Oban, PA37 1QA, United Kingdom

*Anabaena variabilis* ATCC 29413	CP000117	98	2457	92	0.0	It has been studied extensively for over 40 years and is the strain of choice for many laboratories throughout the world

*Nostoc* sp. PCC 7120	BA000019	98	2453	92	0.0	Complete genomic sequence of the filamentous nitrogen-fixing cyanobacterium *Anabaena* sp. (*Nostoc*) strain PCC 7120 is available

*Nostoc commune* NC1 clone 10	EU586726	98	2441	91	0.0	John Carroll University, 20700, North Park Boulevard, University Heights, OH 44118, USA

*Nostoc commune* NC1 clone M2	EU586725	98	2441	91	0.0	John Carroll University, 20700, North Park Boulevard, University Heights, OH 44118, USA

*Nostoc commune* NC5 clone 10	EU586727	98	2437	91	0.0	Biology, John Carroll University, 20700, North Park Boulevard, University Heights, OH 44118, USA

*Nostoc commune* NC1	EU784149	98	2428	91	0.0	Nostoc commune NC1 was isolated from soil (subaerophyt) in Třeboň/Czech republic in 2006.

*Nostoc commune* NC5 clone 11	EU586728	98	2423	91	0.0	Biology, John Carroll University, 20700, North Park Boulevard, University Heights, OH 44118, USA

*Nostoc ellipsosporum* CCAP 1453/15	HE975023	94	2399	92	0.0	Scottish Association for Marine Science, Molecular and Microbial Biology, Dunstaffnage Marine Laboratory, Oban PA37 1QA, United Kingdom

*Nostoc* cf. *punctiforme* Bashkir clone 6A	EU586732	96	2378	92	0.0	John Carroll University, 20700, North Park Boulevard, University Heights, OH 44118, USA

*Calothrix* sp. HA4356-MV2 clone p8i	JN385289	98	2361	90	0.0	Cave wall scraping, Maniniholo Cave near Haena, USA: Kauai, Hawaii

*Calothrix* sp. HA4340 LM2	KF417425	98	2361	90	0.0	Cave, USA: Kauai, Hawaii, Maniniholo Cave

*Anabaena* sp. CCAP 1403/4A	HE975015	93	2360	92	0.0	Scottish Association for Marine Science, Molecular and Microbial Biology, Dunstaffnage Marine Laboratory, Oban PA37 1QA, United Kingdom

*Nostoc muscorum* CCAP 1453/8	HF678508	93	2358	92	0.0	United Kingdom: Scotland

*Calothrix* sp. HA4356-MV2 clone p8i	JN385289	98	2356	90	0.0	Cave wall scraping, Maniniholo Cave near Haena, USA: Kauai, Hawaii

*Cylindrospermum moravicum* CCALA 993 clone operon 1	KF052607	97	2331	94	0.0	Cave sediment, Czech Republic: Amaterska Cave, South Moravia

*Nostoc punctiforme* PCC 73102	CP001037	97	2331	91	0.0	A symbiont from a cycad

*Nostoc* sp. *Peltigera malacea* DB3992 cyanobiont	JX219483	97	2322	91	0.0	Cyanobiont of lichenized fungi *Peltigera malacea*, Iceland

*Nostoc muscorum* CCAP 1453/20	HF678506	95	2318	91	0.0	Scottish Association for Marine Science, Molecular and Microbial Biology, Dunstaffnage Marine Laboratory, Oban PA37 1QA, United Kingdom

*Nostoc* sp. 10Dp66E	JQ259187	98	2318	90	0.0	From association with *Dynamena pumila* L., White Sea, Russia

*Cylindrospermum catenatum* CCALA 999 clone operon 1	KF052615	97	2309	94	0.0	Soil, Slovakia: forest above Stara Brzotinska Cave, Slovak Karst

*Trichormus anomalus* HA4352 LM2	KF417426	96	2309	92	0.0	Cave, USA: Kauai, Hawaii, Maniniholo Cave

*Cylindrospermum* sp. HA4236-MV2 clone p4	JN385290	98	2309	89	0.0	Taro field, Makiki Nature Center, USA: Oahu, Hawaii

*Cylindrospermum catenatum* CCALA 996 clone operon 1	KF052611	97	2307	94	0.0	Soil, Czech Republic: Amaterska Cave, South Moravia

*Tolypothrix campylonemoides* FI5-MK38 clone p10D	JQ083654	98	2305	90	0.0	Sand, USA: Fort Irwin NTC, San Bernardino Co., California

*Cylindrospermum catenatum* CCALA 990 clone operon 1	KF052601	95	2302	94	0.0	Soil, Czech Republic: Benešov nad Černou, South Bohemia

*Spirirestis rafaelensis* WJT-71-NPBG6 clone p1B	JQ083656	98	2300	90	0.0	Joshua Tree National Park, USA: Joshua Tree Forest, San Bernardino Co., California

*Cylindrospermum badium* CCALA 1000 clone operon 1	KF052616	97	2298	94	0.0	Reclaimed coal mine soil, USA: Pyramid State Recreation Area, Illinois

*Cylindrospermum catenatum* CCALA 991 clone operon 1	KF052603	97	2293	94	0.0	Soil, Czech Republic: Most Region, North Bohemia

*Spirirestis rafaelensis *WJT-71-NPBG6 clone p1A	JQ083655	98	2291	89	0.0	Joshua Tree National Park, USA: Joshua Tree Forest, San Bernardino Co., California

*Nostoc* sp. *Peltigera membranacea* cyanobiont N6	JX975209	97	2289	91	0.0	Symbiont of *Peltigera membranacea* lichen, Iceland

*Cylindrospermum pellucidum* CCALA 992 clone operon 1	KF052605	97	2287	94	0.0	Cave sediment, Slovakia: Dlha chodba in Domica Cave system, Slovak Karst

*Hassallia* sp. EM2-HA1 clone p4B	HQ847555	98	2282	89	0.0	Soil, Mojave National Preserve, USA: San Bernardino Co., California

*Tolypothrix tenuis* f. *terrestris* UFS-BI-NPMV-1A2-F06 clone p13E	JQ083651	98	2277	89	0.0	Arid soil after a burn, foothills of the Onaquee Mts. USA: Utah

*Nostoc *cf.* commune* 257-16	HQ877826	96	2271	90	0.0	Subaerial, on Bonampak's archeological building walls, Mexico: Chiapas

*Hassallia* sp. CNP3-B3-C04 clone p5D	HQ847556	98	2271	89	0.0	Soil, Needles District, Virginia Park, Canyonlands National Park, USA: San Bernardino Co., California

Uncultured cyanobacterium clone Emix3.12	JX887892	92	2269	91	0.0	Freshwater microbial mat Konstanz, Germany

*Cylindrospermum muscicola* SAG 44.79 clone operon 1	KF111150	96	2268	93	0.0	Soil France: Gif-Sur-Yvette, Ile-de-France Region

*Tolypothrix tenuis* f. terrestris UFS-BI-NPMV-1A2-F06 clone p13F	JQ083652	98	2268	89	0.0	Arid soil after a burn, foothills of the Onaquee Mts. USA: Utah

*Hassallia* sp. CM1-HA11 clone p8A	JQ083650	98	2268	89	0.0	Sandy loam near gypsum mine, USA: Clark Mountains, San Bernardino Co., California

*Hassallia* sp. CM1-HA08 clone p7B	JQ083648	98	2268	89	0.0	Sandy loam near gypsum mine, USA: Clark Mountains, San Bernardino Co., California

*Nostoc* cf. *commune* 257-20	HQ877827	94	2266	91	0.0	Biofilms of N. cf. commune were collected at Bonampak archeological area in 2008 from two sites on the building walls (Chiapas, Mexico).

*Tolypothrix campylonemoides* FI5-MK38 clone p10A	JQ083653	98	2266	89	0.0	Sand USA: Fort Irwin NTC, San Bernardino Co., California

*Hassallia* sp. CM1-HA08 clone p7F	HQ847554	98	2266	89	0.0	Soil, Clark Mountains, near gypsum mine USA: San Bernardino Co., California

*Desmonostoc* sp. HA7617 LM4	KF417429	96	2241	90	0.0	USA: Kauai, Hawaii, Waikapalae Cave

*Camptylonemopsis* sp. HA4241-MV5 clone B2-3 + p4	JN385292	96	2223	93	0.0	Moleka stream USA: Oahu, Hawaii

*Anabaena circinalis* 33-10 isolate	EF634474	98	2199	88	0.0	Ohau Channel, New Zealand

*Tolypothrix* sp. PCC 7504 isolate DBSU 18	FJ660999	90	2194	92	0.0	Freshwater Aquarium, Sweden

*Nostoc* sp. UAM 307	HM623782	70	2111	98	0.0	Rock surface of calcareous river Spain: Matarranya River, Teruel, East Spain

*Rivularia* sp. 1PA23	FJ660980	92	2021	88	0.0	Pozas Azules I, Mexico Microbialite freshwater

*Nostoc* sp. 8964:3	AM711541	66	2006	99	0.0	Host is *Gunneraprorepens* (Angiospermae), New Zealand

*Rivularia* sp. 1PA21	FJ660978	92	2004	88	0.0	Institute of Ecology, UNAM (Mexico)

*Nostoc linckia* var. *arvense* IAM M-30	AB325907	65	1999	99	0.0	Cultivated samples from the Institute of Molecular Biosciences at the University of Tokyo

*Aphanizomenon ovalisporum* ILC-164	JF768745	92	1988	93	0.0	Lake Kinneret, Israel; Banker et al., 1997 [5]

*Nostoc muscorum* Ind33	HM573462	65	1988	99	0.0	Paddy field, India: Agricultural Farms, Banaras Hindu University, Varanasi, Uttar Pradesh

**Table 6:** BLAST results obtained by querying the *nifH* gene of *Nostoc *sp.* PTV* with GenBank and geographical and ecological origins of the hits.

Closest GenBank relative	GenBank number	Query coverage %	Score %	Identity %	*E* value	Origin of the strain and reference
*Nostoc muscorum* UTAD N213	GQ443450.1	100	612	99	7*e* − 172	Rice paddy in Mondego River Basin, Portugal

*Nostoc muscorum *clone CC1090A1	AY221814.1	94	576	99	5*e* − 161	Ocean Sciences Department, University of California, Santa Cruz, CA 95064, USA

*Nostoc commune* (UTEX 584)	L23514.1	99	565	97	9*E* − 158	Scotland

*Nostoc* sp. UAM 362	JQ514115.1	99	553	96	5*e* − 154	Rock surface of calcareous river with brackish water, Spain: Muga, Girona (Northeast Spain)

*Nostoc* sp. Baikal	JN887856.1	99	517	94	4*e* − 143	Nitrogen-fixing cyanobacteria from Lake Baikal

*Nodularia spumigena* AV1	GQ456132.1	99	511	93	2*e* − 141	The surface waters of the Baltic Sea, Stockholm, Sweden

*Tolypothrix* sp. UAM 379	JQ514114.1	99	462	90	8*E* − 127	Rock surface of calcareous river with brackish water, Spain: Muga, Girona (Northeast Spain)

*Tolypothrix* sp. UAM 378	JQ514113.1	99	462	90	8*E* − 127	Rock surface of calcareous river with brackish water, Spain: Muga, Girona (Northeast Spain)

*Tolypothrix* sp. UAM 415	JQ514120.1	99	453	89	4*E* − 124	Rock surface of calcareous river with brackish water, Spain: Muga, Girona (Northeast Spain)

*Tolypothrix* sp. UAM 413	JQ514119.1	99	453	89	4*E* − 124	Rock surface of calcareous river with brackish water, Spain: Muga, Girona (Northeast Spain)

*Nostoc* PCC 6720	Z31716.1	99	430	88	4*E* − 117	*Nostoc* PCC 6720 was previously known as *Anabaenopsis circularis*. This is a freshwater species

*Anabaena* sp. L-31	L04499.1	99	426	88	5*E* − 116	The filamentous, heterocystous, nitrogen-fixing freshwater cyanobacterium

*Anabaena* PCC7120	J05111.1	99	426	88	5*E* − 116	http://wiki.annotation.jp/Kazusa:CyanoBase:Anabaena_sp._PCC_7120

*Nostoc* sp. UAM 367	JQ514116.1	100	423	87	7*E* − 115	Rock surface of calcareous river with brackish water, Spain: Muga, Girona (Northeast Spain)

*Anabaena siamensis* TISTR 8012	DQ176436.2	100	414	87	3*E* − 112	*Anabaena siamensis* is a filamentous heterocystous nitrogen-fixing cyanobacterium which originally was isolated from a rice paddy field in Thailand

*Mastigocladus laminosus* CCMEE 5324	EF570558.1	100	414	87	3*E* − 112	The cosmopolitan thermophilic cyanobacterium *Mastigocladus laminosus* from the University of Oregon's Culture Collection of Microorganisms from Extreme Environments (CCMEE)

*Fischerella* UTEX1931	U49514.1	100	414	87	3*E* − 112	Thermophilic cyanobacterium (synonym: *Mastigocladus laminosus*)

*Anabaena sphaerica* UTEX “B 1616”	DQ439648.1	99	408	87	1*E* − 110	Department of Chemistry and Chemical Engineering, University of Sheffield, Mappin Street, Sheffield, South Yorkshire S1 3JD, United Kingdom

*Anabaena* sp. A2	AF124377.1	99	408	87	1.*E* − 110	Molecular Evolution, BMC, Uppsala University, Husargatan 3, 751 24 Uppsala, Sweden

*Anabaena azollae *	L34879.1	99	435	88	1*E* − 118	*Anabaena azollae* 1a, a putative symbiont of *Azolla caroliniana *

*Anabaena variabilis *	U89346.1	99	435	88	1*E* − 118	*Anabaena variabilis ATCC* 29413 is a filamentous cyanobacterium that produces heterocysts and fixes nitrogen under a variety of environmental conditions
